# Temperature-Dependent Kinetic Study of the Reactions of Hydrogen Atoms with H_2_S and C_2_H_4_S

**DOI:** 10.3390/molecules28237883

**Published:** 2023-11-30

**Authors:** Yuri Bedjanian

**Affiliations:** Institut de Combustion, Aérothermique, Réactivité et Environnement (ICARE), CNRS, 45071 Orléans, France; yuri.bedjanian@cnrs-orleans.fr; Tel.: +33-23-825-5474

**Keywords:** hydrogen atom, hydrogen sulfide, thiirane, H_2_S, C_2_H_4_S, kinetics, rate constant

## Abstract

A discharge-flow reactor combined with modulated molecular beam mass spectrometry technique was employed to determine the rate constants of H-atom reactions with hydrogen sulfide and thiirane. The rate constants for both reactions were determined at a total pressure of 2 Torr from 220 to 950 K under pseudo-first-order conditions by monitoring either consumption of H atoms in excess of H_2_S (C_4_H_4_S) or the molecular species in excess of atomic hydrogen. For H + H_2_S reaction, a suggested previously strong curvature of the Arrhenius plot was confirmed: *k*_l_ = 8.7 × 10^−13^ × (*T*/298)^2.87^ × exp(−125/*T*) cm^3^ molecule^−1^ s^−1^ with a conservative uncertainty of 15% at all temperatures. Non-Arrhenius behavior was also observed for the reaction of H-atom with C_2_H_4_S, with the experimental rate constant data being best fitted to a sum of two exponential functions: *k*_2_ = 1.85 × 10^−10^ exp(−1410/*T*) + 4.17 × 10^−12^ exp(−242/*T*) cm^3^ molecule^−1^ s^−1^ with an independent of temperature uncertainty of 15%.

## 1. Introduction

The present work reports temperature-dependent measurements of rate constants for two elementary reactions involving hydrogen atoms, with H_2_S and C_2_H_4_S. The H-atom reaction with H_2_S is of importance in combustion chemistry and industrial processes, and is included in a detailed chemical mechanism to describe reactions in the H_2_–S_2_–H_2_S system [1]:

H + H_2_S → SH + H_2_ (1)

The temperature dependence of the rate constant of reaction (1), which proceeds through the abstraction of a hydrogen atom, is also of theoretical interest, in particular for assessing the effect of quantum tunneling [2,3]. Reaction (1) has been intensively studied over the past few decades, both experimentally [4,5,6,7,8,9,10,11,12,13,14,15] and theoretically [2,3,13,15,16]. Although there is now some experimental and theoretical evidence for the curvature of the Arrhenius plot for reaction (1), it has never actually been observed experimentally in any single study conducted over a sufficiently wide temperature range. All previous experimental work was carried out over a limited temperature range, and “unmodified” Arrhenius expressions were reported for the reaction rate constant. One of the aims of this work was to provide experimental evidence of the curvature of the Arrhenius dependence of *k*_1_ within the framework of one study through rate constant measurements over an extended temperature range, *T* = 220–950 K.

It is known that reactions of C_2_H_4_S (thiirane, ethylene sulfide) with various atoms are rapid and proceed through S-atom abstraction, leading to almost stoichiometric production of ethylene and sulfur-containing radicals [17,18,19,20]. This allows the desulfurization reactions of thiirane to be used in laboratory research both as sources of radicals and as scavengers of unwanted active species. The reaction of C_2_H_4_S with hydrogen atoms leads to the formation of SH (an important intermediate in atmospheric and combustion chemistry [21,22]), and can be used as an alternative source of SH radicals to those involving H_2_S [23].

H + C_2_H_4_S → SH + C_2_H_4_ (2)

The kinetic information on reaction (2) is rather scarce and uncertain. Only two measurements of the reaction rate constant were reported, by Yokota et al. [24] at *T* = 300–425 K using the relative rate method, and in absolute measurements by Lee et al. from 223 to 423 K [20]. The activation energies reported in two studies are very close; however, the absolute values of the reaction rate constant differ by a factor of 3. The objective of the present work was to provide new measurements of the rate constant over an extended temperature range, *T* = 220–950 K.

## 2. Results and Discussion

### 2.1. Reaction H + H_2_S

#### 2.1.1. Measurements of the Reaction Rate Constant

All measurements were carried out under flow conditions at nearly 2 Torr of total pressure of Helium, and with detection of the gas phase species involved using mass spectrometry [23].

The reaction rate constant was determined under pseudo-first-order conditions either from H_2_S decays ([H_2_S]_0_ ≤ 4 × 10^11^ molecule cm^−3^) in excess of hydrogen atoms, or from the kinetics of H-atom consumption ([H]_0_ ≤ 2 × 10^11^ molecule cm^−3^) monitored in excess of hydrogen sulfide. The concentrations of the excess reactants were varied between 0.18 and 6.64 × 10^13^ molecule cm^−3^ for H-atom, and between 0.85 and 44.7 × 10^13^ molecule cm^−3^ for [H_2_S] (see Table 1). The linear flow velocity in the reactor was in the range of 1025–3400 cm s^−1^.

**Figure 1:**
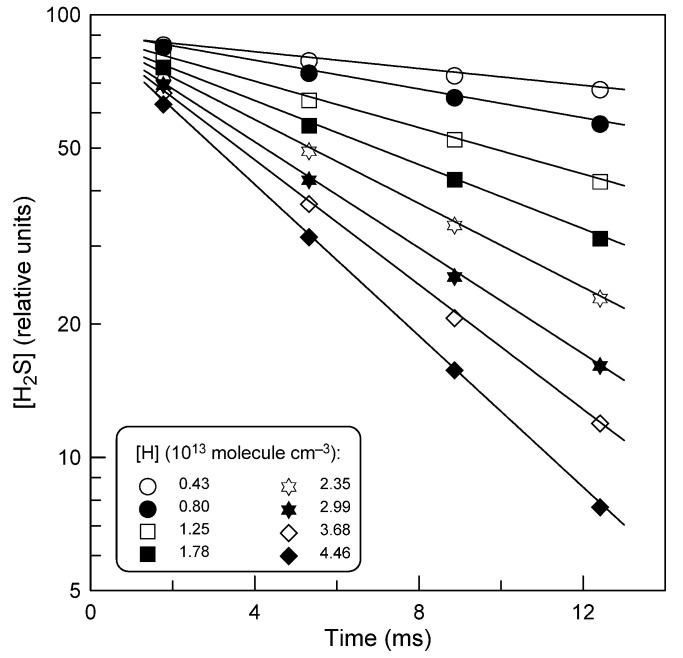
Reaction H + H_2_S: example of H_2_S decays observed with different concentrations of H atoms at *T* = 575 K.

Examples of H_2_S decays observed at different excess concentrations of hydrogen atoms are shown in Figure 1. The consumption of the excess reactant, H atoms, along the reaction zone did not exceed a few percent (mainly due to the wall loss, *k*_w_ < 10 s^−1^). In all cases, the average concentration of H was used in the calculations. The concentration of H_2_S decays exponentially, [H_2_S] = [H_2_S]_0_ × exp(−*k*_1_^′^ × t), where *k*_1_^′^ = *k*_1_[H] is the pseudo-first-order rate constant of H_2_S loss.

**Figure 2:**
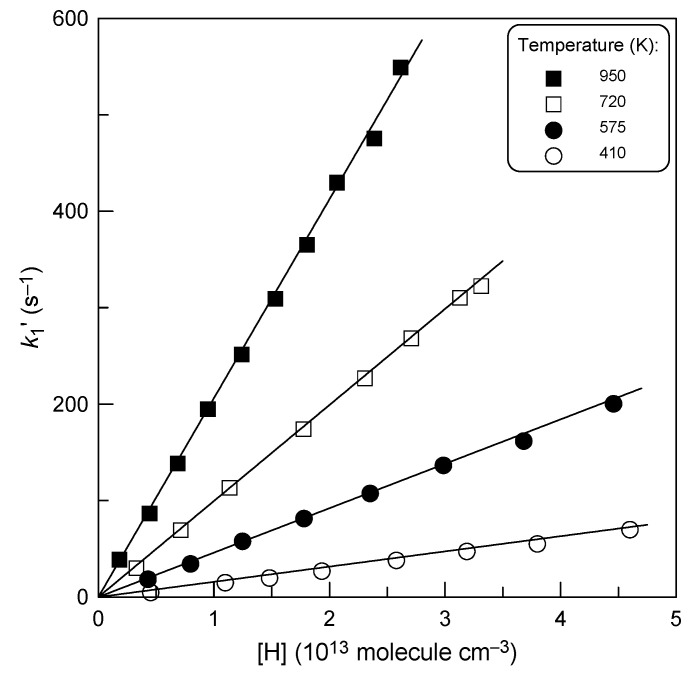
Reaction H + H_2_S: dependence of the pseudo-first-order rate constant, *k*_1_^′^ = *k*_1_[H], on the concentration of H atoms at different temperatures.

The pseudo-first-order rate constants, *k*_1_^′^, determined from the slopes of the straight lines like those in Figure 1, are shown in Figure 2, as a function of H-atom concentration.

One can note that the intercepts in Figure 2 are close to zero, which is consistent with the observed lack of H_2_S consumption in the absence of hydrogen atoms in the reactor. Diffusion corrections were applied to all the measured values of *k*_1_^′^ in order to take into account the axial and radial gradient of H_2_S concentration in the flow tube [25]. Corrections were generally less than 3%, and only at *T* = 950 K were they somewhat higher, reaching 7%. The slopes of the straight lines in Figure 2 provide the bimolecular rate constants at respective temperatures, which are summarized in Table 1.

**Figure 3:**
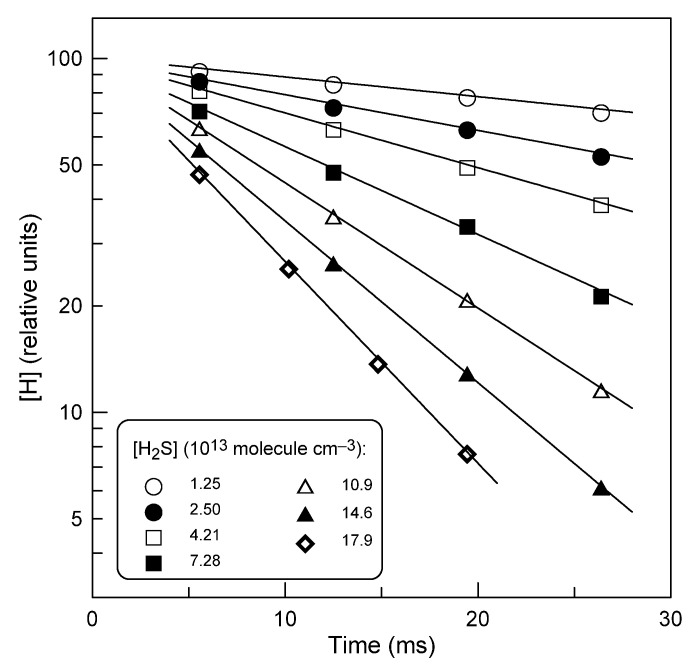
Reaction H + H_2_S: typical H-atom decays observed in the presence of different concentrations of H_2_S at *T* = 325 K.

In the second series of experiments, the kinetics of H-atom consumption in an excess of hydrogen sulfide was recorded. Figure 3 displays typical exponential decays of [H] with time, [H] = [H]_0_ × exp(−*k*_1_^′^×t), where *k*_1_^′^ = *k*_1_[H_2_S] + *k*_w_ is the pseudo-first-order rate constant, with *k*_w_ representing the heterogeneous loss of H atoms.

**Figure 4:**
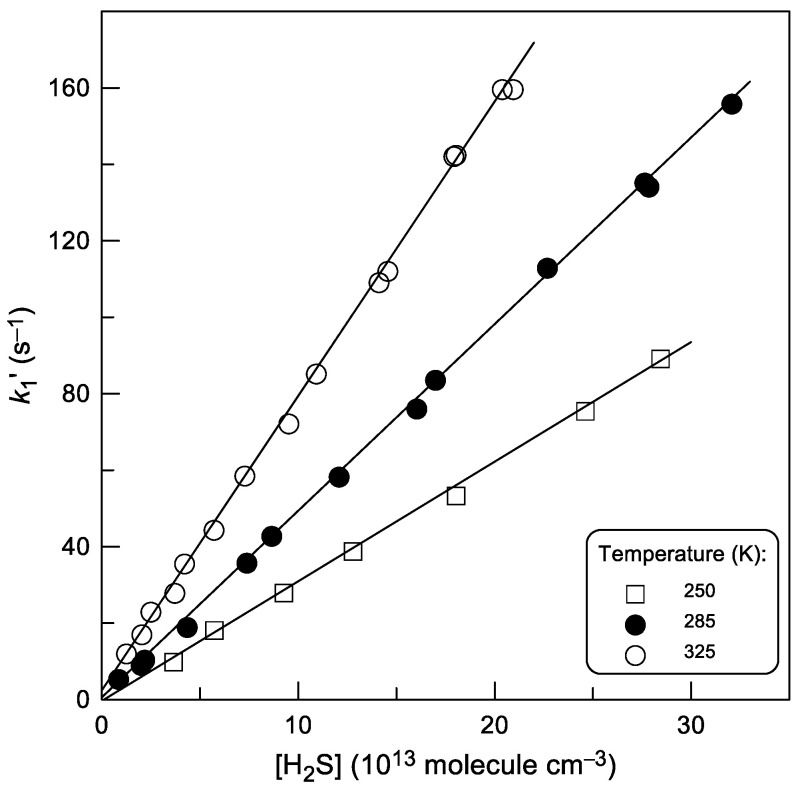
Reaction H + H_2_S: dependence of the pseudo-first order rate constant, *k*_1_^′^ = *k*_1_[H_2_S] + *k*_w_, on the concentration of H_2_S at different temperatures.

Examples of the typical second-order plots observed at different temperatures are shown in Figure 4. The low intercept values of the plots in Figure 4 are in agreement with the wall loss rate of H atoms, *k*_w_ < 10 s^−1^, measured in the absence of H_2_S in the reactor. Diffusion corrections applied to the measured values of *k*_1_^′^ did not exceed 10%. Final values of *k*_1_ determined in this series of experiments are presented in Table 1. The combined uncertainty on *k*_1_ was estimated to be around 15% for both series of experiments by adding in quadrature statistical error (≤2%) and those on the measurements of the absolute concentration of H_2_S (≈7%), (H) (≈10%), flows (3%), pressure (2%), and temperature (1%).

Reaction (1) produces SH radicals, and H-atom kinetics can be potentially impacted by the fast secondary reaction:

H + SH → S + H_2_ (3)

The rate constant of reaction (3) is not well known. Measurements of *k*_3_ available in the literature are scattered between 1.1 × 10^−11^ and 4.15 × 10^−11^ cm^3^ molecule^−1^ s^−1^ at room temperature [4,8,10,26]. Although this secondary reaction is quite fast, its impact on the measurements of *k*_1_ can be considered negligible due to the low initial concentrations of H atoms used ([H]_0_ ≤ 2 × 10^11^ molecule cm^−3^).

#### 2.1.2. Comparison with Previous Data

**Figure 5:**
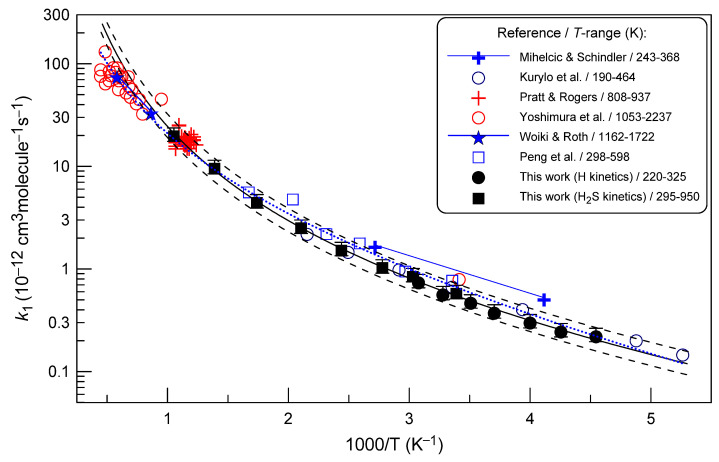
Temperature dependence of the rate constant of reaction (1). Uncertainties shown for the present measurements of *k*_1_ correspond to estimated total uncertainty of 15%.

Figure 5 shows the temperature-dependent data available for *k*_1_. As noted in the Introduction section and as can be seen in Figure 5, the rate constant for reaction (1) is relatively well established.

The most reliable measurements of *k*_1_ at room temperature [8,10,11,12] (not shown in Figure 5 for clarity) are in good agreement with each other. Bradley et al. [8] measured *k*_1_ in a flow reactor using electron spin resonance for detection of hydrogen atoms: *k*_l_ = 8.3 × 10^−13^ cm^3^ molecule^−1^ s^−1^. The same value was reported for *k*_1_ by Nicholas et al. [10] who monitored the concentrations of SH and S_2_ intermediates upon decomposition of H_2_S in a pulsed radio-frequency discharge and derived *k*_1_ from computer simulation of SH and S_2_ profiles. Husain and Slater [11] reported an absolute value of *k*_l_ = (8.6 ± 0.5) × 10^−13^ cm^3^ molecule^−1^ s^−1^, determined via the pulsed photolysis resonance fluorescence method. Finally, Clyne and Ono [12] measured *k*_l_ = (7.41 ± 0.39) × 10^−13^ cm^3^ molecule^−1^ s^−1^, employing resonance fluorescence method in a discharge flow system. It can be noted that the present value of *k*_l_ measured around room temperature, e.g., (6.0 ± 0.9) × 10^−13^ cm^3^ molecule^−1^ s^−1^ at *T* = 295 K, is somewhat lower than these previous measurements.

The first temperature-dependent measurement of the rate constant for reaction (1) was conducted by Mihelcic and Schindler [5], who used a discharge flow system combined with ESR detection of hydrogen atoms. The following Arrhenius equation was reported: *k*_l_ = (1.7 ± 0.1) × 10^−11^ exp(−845 ± 25)/*T*] cm^3^ molecule^−1^ s^−1^ at *T* = 243–368 K. Kurylo et al. [6], using flash photolysis coupled with resonance fluorescence, carried out absolute measurements of *k*_l_ over a temperature range of 190–464 K, and reported *k*_l_ = (1.29 ± 0.15) × 10^−11^ exp[ −(860 ± 30)/*T*] cm^3^ molecule^−1^ s^−1^. At high temperatures (*T* > 800 K), the rate constant of reaction (1) has been determined in three studies [9,13,14]. Pratt and Rogers [9] in their mass spectrometric study of the early stages of the exchange reaction in H_2_S/D_2_ mixture deduced values of *k*_l_ at *T* = 808–937 K, which are shown in Figure 5. Yoshimura et al. [13] and Woiki and Roth [14] studied reaction (1) behind reflected shock waves, applying atomic resonance absorption spectroscopy for the measurements of H-atom concentration, and reported *k*_l_ = 3.2 × 10^−10^ exp(−2491/*T*) at *T* = 1053–2237 K and *k*_l_ = 4.15 × 10^−10^ exp(−2890/*T*) cm^3^ molecule^−1^ s^−1^ at *T* = 1160–1722 K, respectively. As one can see in Figure 5, the results of these high temperature measurements are in good agreement. Finally, in the most recent study of the reaction (1) [15], *k*_1_ was measured via the flash photolysis resonance fluorescence technique and found to be: *k*_l_ = 6.6 × 10^−11^ exp(−1347/*T*) cm^3^ molecule^−1^ s^−1^ at T = 298–598 K.

The present measurements of *k*_l_ are in very good agreement with those of Kurylo et al. [6], Pratt and Rogers [9], and, in the range of experimental uncertainty, with the data from Peng et al. [15]. The *k*_l_ values measured by Mihelcic and Schindler [5] are a bit higher than others. Rommel and Schiff [7] suggested that the *k*_l_ values reported by Michelcic and Schindler [5] may have been overestimated. In fact, the values of *k*_l_ were derived from decays of H atoms under experimental conditions, where initial H_2_S concentration was comparable to that of hydrogen atoms. In addition, secondary reaction (3), which should operate under such conditions, was not taken into account.

The present data for *k*_l_ obtained in a wide temperature range clearly show a well-pronounced curvature of the Arrhenius plot. The fit of the current data with the modified Arrhenius expression (the continuous black line in Figure 5) yields

*k*_l_ = 8.7 × 10^−13^ × (*T*/298)^2.87^ × exp(−125/*T*) cm^3^ molecule^−1^ s^−1.^

This expression for *k*_l_ is recommended from the present study in the temperature range 220–950 K, with a conservative (independent of temperature) overall 2σ uncertainty of 15%. The black dashed lines in Figure 5 represent the deviations from this equation by a factor of 1.3, showing that practically all existing data (except at the highest temperatures) fall into this range.

It should be noted that the curvature of the Arrhenius plot is observed experimentally for the first time, although it was suggested in some previous studies. All previous experimental work was carried out over a limited temperature range, and resulted in “unmodified” Arrhenius expressions for *k*_l_. Yoshimura et al. [13] based on their measurements of *k*_l_ at room and high temperatures (Figure 5) suggested a strong non-Arrhenius dependence of *k*_l_ on temperature, which was explained by a conventional transition-state theory combined with ab initio calculations. Peng et al. [15], combining their experimental data (*T* = 298–598 K) with those available in the literature, reported *k*_l_ = 5.8 × 10^−17^ × *T*^1.94^ exp(−455/*T*) cm^3^ molecule^−1^ s^−1^ (blue dotted line in Figure 5). This expression reasonably (within 25%) describes the *k*_l_ data from the present work. The theoretical analysis carried out by the authors also supported the significant curvature of the Arrhenius plot. The experimentally observed non-Arrhenius temperature dependence of *k*_l_ was also reasonably reproduced by the recent quantum dynamics calculations of Qi et al. [2], concluding that the non-Arrhenius behavior is caused by the pronounced quantum tunneling. The calculations of Qi et al. [2] showed that both abstraction and exchange mechanisms are important for the H + H_2_S reaction. Unfortunately, the available experimental data do not make it possible to define the contribution of these two mechanisms as a function of temperature.

### 2.2. Reaction H + C_2_H_4_S

#### 2.2.1. Measurements of the Reaction Rate Constant

**Figure 6:**
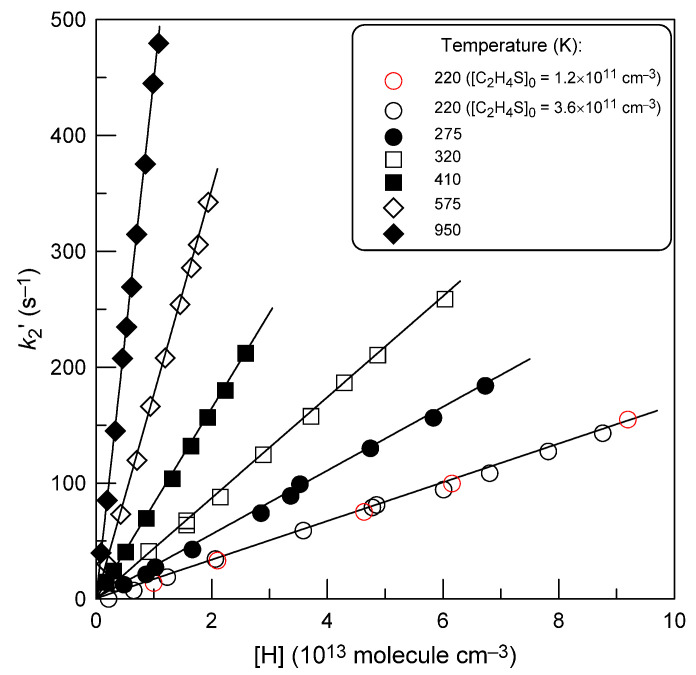
Reaction H + C_2_H_4_S: pseudo-first-order rate constant, *k*_2_^′^ = *k*_2_[H], as a function of the concentration of H atoms at different temperatures.

**Figure 7:**
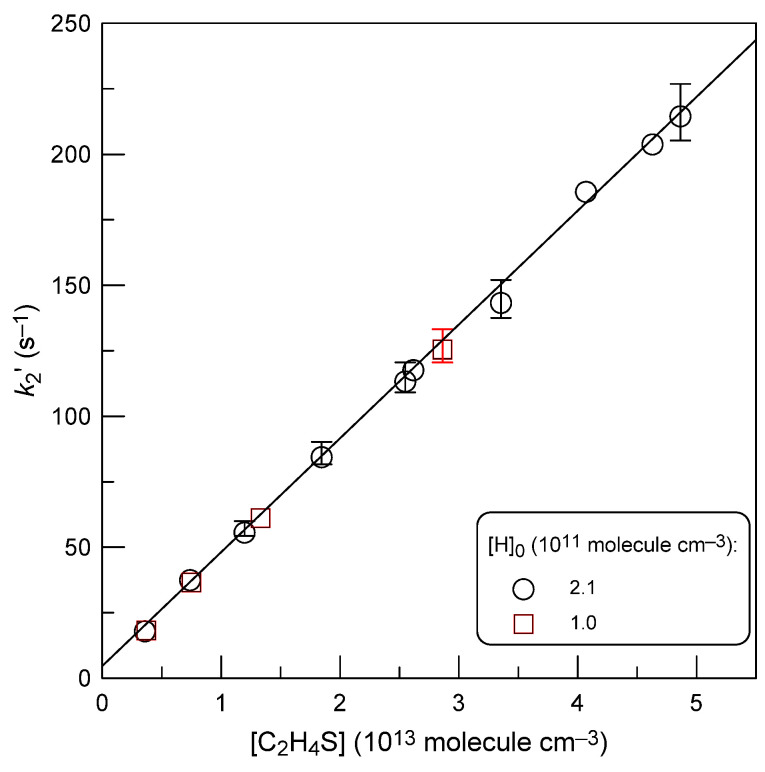
Reaction H + C_2_H_4_S: dependence of the pseudo-first-order rate constant, *k*_2_^′^ = *k*_2_[C_2_H_4_S] + *k*_w_, on the concentration of C_2_H_4_S at *T* = 325 K measured with two different initial concentrations of H atoms.

A similar experimental protocol described above for reaction (1) was also applied to measure the rate constant of the reaction of hydrogen atoms with thiirane. As in the previous case, the rate constant of reaction (2) was determined under pseudo-first order conditions by two methods: from kinetics of thiirane consumption ([C_2_H_4_S]_0_ ≤ 3.6 × 10^11^ molecule cm^−3^) in excess of hydrogen atoms and from H-atom decays ([H]_0_ ≤ 2.1 × 10^11^ molecule cm^−3^) recorded in excess of C_2_H_4_S. The concentrations of the corresponding excess reactants are shown in Table 2. The flow velocity in the reactor was in the range (1045–3250) cm s^−1^. Examples of second-order plots obtained from C_2_H_4_S and H-atom decays are shown in Figure 6 and Figure 7, respectively. The similarity of the results obtained with different initial concentrations of C_2_H_4_S (Figure 6, *T* = 220 K) and hydrogen atoms (Figure 7) indicates a minor influence of possible secondary chemistry on the measurements of *k*_2_. The final values of *k*_2_ determined at different temperatures from the slopes of the straight lines like those in Figure 6 and Figure 7 are shown in Table 2.

#### 2.2.2. Comparison with Previous Data

**Figure 8:**
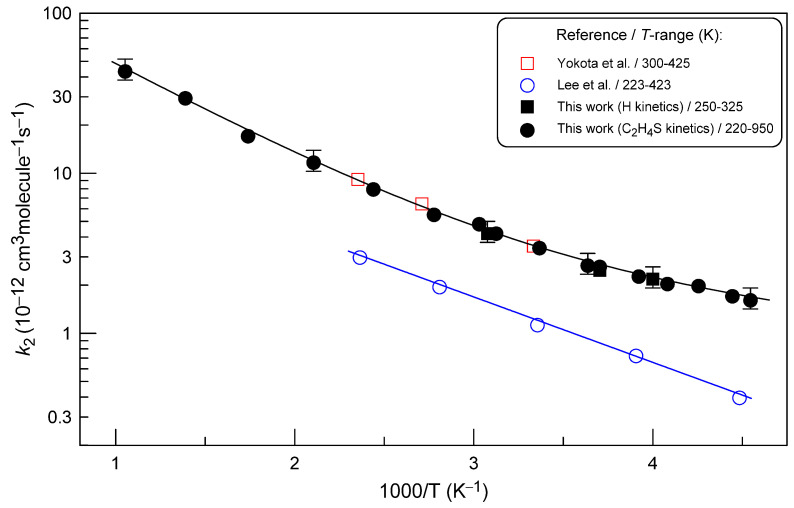
Summary of the measurements of *k*_2_. Uncertainties shown for selected present measurements of *k*_2_ correspond to the estimated total uncertainty of 15%.

The rate constant of the H + C_2_H_4_S reaction is displayed as a function of temperature in Figure 8. The present data on the temperature dependence of *k*_2_ are best represented by the sum of two exponential functions (the continuous black line in Figure 8):

*k*_2_ = 1.85×10^−10^ exp(−1410/*T*) + 4.17×10^−12^ exp(−242/*T*) cm^3^ molecule^−1^ s^−1^

at *T* = 220–950 K (with an estimated conservative uncertainty of 15% at all temperatures).

To our knowledge, only two studies of the reaction are available in the literature. Yokota et al. [24] studied the reaction of thiirane with hydrogen atoms produced from the mercury photosensitization of H_2_. The reaction rate constant was determined relative to that of the H-atom reaction with H_2_S at three temperatures between 300 and 425 K: *k*_2_ = (9.5 ± 1.2) × 10^−11^ exp[−(980 ± 88)/*T*] cm^3^ molecule^−1^ s^−1^. The relative rate data were placed on an absolute basis with the values of *k*_1_ reported by Kurylo et al. [6], which can be considered valid to date (see Figure 5). Lee et al. [20] carried out absolute measurements of *k*_2_ using the flash photolysis–resonance fluorescence technique, and reported *k*_2_ = (2.87 ± 0.12) × 10^−11^ exp[−(945 ± 12)/*T*] cm^3^ molecule^−1^ s^−1^ over a temperature range from 223 to 423 K. The present data for *k*_2_ clearly support the results of the relative rate measurements of Yokota et al. [24], being higher by a factor of 3–4 compared with the absolute measurements of Lee et al. [20] (Figure 8). The reason for such a large discrepancy is difficult to determine at this stage. Perhaps the point is in determining the absolute concentrations of thiirane, which (i) was photolyzed in the study of Lee at al. [20] to generate H atoms, and (ii) is known to decompose during storage.

In the study of Yokota et al. [24], C_2_H_4_, H_2_S, and elemental sulfur were detected as reaction products in accordance with the formation of SH radical and C_2_H_4_ in the primary step, followed by the production of H_2_S and S in the self-reaction of SH radicals:

SH + SH → S + H_2_S (4)

S + C_4_H_4_S → S_2_ + C_2_H_4_ (5)

In this work, we also observed the formation of SH, S and S_2_ under specific conditions (high concentrations of both reactants); however, quantitative measurements were quite difficult, and were not performed.

## 3. Materials and Methods

**Figure 9:**
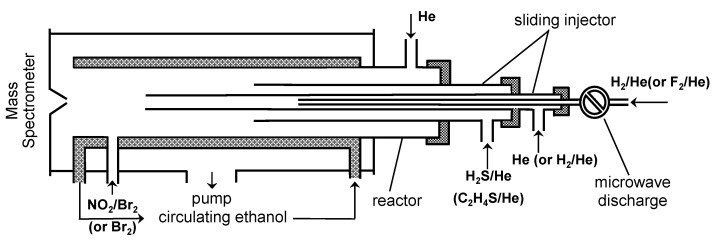
Low-temperature flow reactor: configuration used in the measurements of the rate constants of reactions (1) and (2).

All experiments were performed in a conventional discharge fast-flow reactor coupled with a modulated molecular beam-sampling quadrupole mass spectrometer for the detection of the gas phase species [23]. Two different flow reactors (45 cm length and 2.4 cm i.d.) available in the laboratory were used. The low-temperature flow reactor, a Pyrex tube coated with halocarbon wax with a jacket for the circulation of thermostatically controlled ethanol (Figure 9), covered a temperature range between 220 and 330 K. The high-temperature flow reactor was employed over a temperature range of 295−950 K and consisted of an electrically heated uncoated Quartz tube with water-cooled attachments.

Hydrogen atoms were produced using two methods. The first one employed the microwave discharge of H_2_/He mixtures. In the second method, H atoms were formed in the fast reaction of F atoms with H_2_,

F + H_2_ → H + HF (6)

*k*_6_ = 1.24 × 10^−10^ exp(−507/*T*) cm^3^ molecule^−1^ s^−1^ (*T* = 220–960 K) [27].

In this case, the fluorine atoms were generated in the microwave discharge of F_2_/He mixtures and titrated with an excess of H_2_ in the movable injector (Figure 9). The fraction of F_2_ dissociated in the microwave discharge exceeded 95%. Ensuring that there was no molecular fluorine in the reactor was important in the presence of thiirane, since it was observed that these two stable compounds show high reactivity towards each other.

Low concentrations of hydrogen atoms were monitored using their chemical conversion to stable species HOBr (*m*/*z* = 96/98) upon addition of the NO_2_/Br_2_ mixture 5 cm upstream of the sampling cone of the mass spectrometer (Figure 9). In this configuration, H atoms are converted to HOBr in two successive rapid reactions (7) and (8):

H + NO_2_ → OH + NO (7)

*k*_7_ = (1.47 ± 0.26) × 10^−10^ cm^3^ molecule^−1^ s^−1^ (*T* = 195–2000 K) [28],

OH + Br_2_ → Br + HOBr (8)

*k*_8_ = 2.16 × 10^−11^ exp(207/*T*) cm^3^ molecule^−1^ s^−1^ (*T* = 220–950 K) [29].

The concentrations of NO_2_ and Br_2_ were chosen so that the hydrogen atoms reacted primarily with NO_2_ rather than with Br_2_ in reaction (9):

H + Br_2_ → Br + HBr (9)

*k*_9_ = 7.06 × 10^−11^ (*T*/298)^0.88^ exp(182/*T*) cm^3^ molecule^−1^ s^−1^ (*T* = 220–950 K) [30].

In experiments with high concentrations of hydrogen atoms (kinetics of H_2_S, and C_2_H_4_S consumption in excess of H), they were detected at m/z = 80/82 (HBr^+^) after being transformed to HBr in reaction (9).

The absolute calibration of the mass spectrometer for HOBr was accomplished using reaction (8) in excess of Br_2_, and relating the consumed fraction of Br_2_ to the concentration of HOBr produced: [HOBr] = Δ[Br_2_]. Absolute HBr concentrations were determined using two methods. In the first method, the absolute concentration of HBr was calculated from the flow rate of a manometrically prepared HBr/He mixture. The second method was the chemical transformation of the H atom into HBr in reaction (9), linking the consumed fraction of Br_2_ and the concentration of HBr formed: [HBr] = Δ[Br_2_]. The results of the two methods coincided within a few percent. The absolute concentrations of other stable species (Br_2_, NO_2_, F_2_, H_2_, H_2_S and C_2_H_4_S) in the reactor were calculated from their flow rates, obtained from the measurements of the pressure drop of their monometrically prepared mixtures in He stored in calibrated volume flasks.

The purities of the gases used were as follows: He > 99.999% (Alphagaz); Br_2_ > 99.99% (Aldrich); F_2_, 5% in helium (Alphagaz); HBr > 99.8% (Praxair); H_2_ > 99.998% (Alphagaz); NO_2_ > 99% (Alphagaz); H_2_S > 99.5% (Alphagaz); C_2_H_4_S (Merck), 98%.

## 4. Conclusions

In this work, we have investigated the kinetics of the reactions of H atoms with hydrogen sulfide and thiirane, using a discharge flow reactor combined with mass spectrometry. For the H + H_2_S reaction, previously assumed on the basis of experimental and theoretical data, the strong curvature of the Arrhenius plot was confirmed via measurements of the reaction rate constant over an extended temperature range (220–950 K). Similar curved Arrhenius dependence was also observed for reaction of H atoms with C_2_H_4_S, which was studied at *T* = 220–950 K (and for the first time at *T* > 425 K). The reaction was found to be fast enough to be used as an alternative (free of H_2_S) source of SH radicals (an important intermediate in combustion and atmospheric chemistry) in laboratory studies.

## Figures and Tables

**Table 1:** Experimental conditions and results of the measurements of the rate constant of reaction (1).

*T* (K)	[Excess Reactant] *^a^*	*k*_1_ *^b^*	Reactor Surface *^c^*	Method *^d^*
220	6.51–24.2	0.24 ± 0.01	HW	H kinetics
235	4.80–28.8	0.26 ± 0.01	HW	H kinetics
250	3.66–28.4	0.31 ± 0.01	HW	H kinetics
270	1.60–44.7	0.39 ± 0.01	HW	H kinetics
285	0.85–27.8	0.49 ± 0.01	HW	H kinetics
295	0.66–4.37	0.60 ± 0.01	Q	H_2_S kinetics
305	1.09–19.7	0.59 ± 0.01	Q	H kinetics
325	1.25–20.4	0.77 ± 0.02	HW	H kinetics
330	0.46–6.64	0.88 ± 0.02	HW	H_2_S kinetics
360	0.62–5.58	1.07 ± 0.02	Q	H_2_S kinetics
410	0.45–4.60	1.58 ± 0.02	Q	H_2_S kinetics
475	0.57–5.87	2.61 ± 0.03	Q	H_2_S kinetics
575	0.43–4.46	4.61 ± 0.08	Q	H_2_S kinetics
720	0.33–3.31	9.96 ± 0.08	Q	H_2_S kinetics
950	0.18–2.61	20.6 ± 0.3	Q	H_2_S kinetics

*^a^* Units of 10^13^ molecule cm^−3^. *^b^* units of 10^−12^ cm^3^ molecule^−1^s^−1^; statistical 2σ uncertainty is given, total estimated uncertainty is 15%. *^c^* HW: halocarbon wax; Q: quartz. *^d^ k*_1_ derived from H_2_S (H_2_S kinetics) or H-atom (H kinetics) decays monitored in excess of H and H_2_S, respectively.

**Table 2:** Experimental conditions and results of the measurements of the rate constant of reaction (2).

*T* (K)	[Excess Reactant] *^a^*	*k*_2_ *^b^*	Reactor Surface *^c^*	Method *^d^*
220	0.21–9.19	1.67 ± 0.04	HW	C_2_H_4_S kinetics
225	0.40–8.82	1.77 ± 0.03	HW	C_2_H_4_S kinetics
235	0.8–7.73	2.05 ± 0.04	HW	C_2_H_4_S kinetics
245	0.26–7.25	2.11 ± 0.03	HW	C_2_H_4_S kinetics
250	0.23–3.86	2.26 ± 0.06	HW	H kinetics
255	0.45–8.28	2.35 ± 0.04	HW	C_2_H_4_S kinetics
270	0.24–4.03	2.56 ± 0.07	HW	H kinetics
270	0.56–6.44	2.7 ± 0.04	HW	C_2_H_4_S kinetics
275	0.48–6.73	2.75 ± 0.07	HW	C_2_H_4_S kinetics
297	0.11–3.84	3.54 ± 0.04	Q	C_2_H_4_S kinetics
320	0.33–6.03	4.36 ± 0.04	HW	C_2_H_4_S kinetics
325	0.36–4.86	4.35 ± 0.08	HW	H kinetics
330	0.30–4.54	4.99 ± 0.06	HW	C_2_H_4_S kinetics
360	0.23–3.34	5.71 ± 0.06	Q	C_2_H_4_S kinetics
410	0.18–2.58	8.23 ± 0.07	Q	C_2_H_4_S kinetics
475	0.15–2.24	12.1 ± 0.1	Q	C_2_H_4_S kinetics
575	0.18–1.94	17.7 ± 0.1	Q	C_2_H_4_S kinetics
720	0.14–1.36	30.5 ± 0.2	Q	C_2_H_4_S kinetics
950	0.09–1.08	44.9 ± 0.1	Q	C_2_H_4_S kinetics

*^a^* Units of 10^13^ molecule cm^−3^. *^b^* units of 10^−12^ cm^3^ molecule^−1^s^−1^; statistical 2σ uncertainty is given; total estimated uncertainty is 15%. *^c^* HW: halocarbon wax; Q: quartz. *^d^ k*_2_ derived from C_2_H_4_S (C_2_H_4_S kinetics) or H-atom (H kinetics) decay monitored in excess of H and C_2_H_4_S, respectively.

## Data Availability

The data supporting reported results are available in this article.
